# A20/TNFAIP3 Increases ENOS Expression in an ERK5/KLF2-Dependent Manner to Support Endothelial Cell Health in the Face of Inflammation

**DOI:** 10.3389/fcvm.2021.651230

**Published:** 2021-05-07

**Authors:** Cleide Angolano, Elzbieta Kaczmarek, Sanah Essayagh, Soizic Daniel, Lynn Y. Choi, Brian Tung, Gabriel Sauvage, Andy Lee, Franciele C. Kipper, Maria B. Arvelo, Herwig P. Moll, Christiane Ferran

**Affiliations:** ^1^The Division of Vascular and Endovascular Surgery and the Center for Vascular Biology Research, Department of Surgery, Beth Israel Deaconess Medical Center and Harvard Medical School, Boston, MA, United States; ^2^The Division of Neurosurgery and the Center for Vascular Biology Research, Department of Surgery, Beth Israel Deaconess Medical Center and Harvard Medical School, Boston, MA, United States; ^3^The Transplant Institute and the Division of Nephrology, Beth Israel Deaconess Medical Center and Harvard Medical School, Boston, MA, United States

**Keywords:** A20/TNFAIP3, endothelial nitric oxidase synthase, inflammation, Kruppel-like factor 2, atherosclerosis, endothelial cell dysfunction

## Abstract

**Rationale:** Decreased expression and activity of endothelial nitric oxide synthase (eNOS) in response to inflammatory and metabolic insults is the hallmark of endothelial cell (EC) dysfunction that preludes the development of atherosclerosis and hypertension. We previously reported the atheroprotective properties of the ubiquitin-editing and anti-inflammatory protein A20, also known as TNFAIP3, in part through interrupting nuclear factor-kappa B (NF-κB) and interferon signaling in EC and protecting these cells from apoptosis. However, A20's effect on eNOS expression and function remains unknown. In this study, we evaluated the impact of A20 overexpression or knockdown on eNOS expression in EC, at baseline and after tumor necrosis factor (TNF) treatment, used to mimic inflammation.

**Methods and Results:** A20 overexpression in human coronary artery EC (HCAEC) significantly increased basal eNOS mRNA (qPCR) and protein (western blot) levels and prevented their downregulation by TNF. Conversely, siRNA-induced A20 knockdown decreased eNOS mRNA levels, identifying A20 as a physiologic regulator of eNOS expression. By reporter assays, using deletion and point mutants of the human eNOS promoter, and knockdown of eNOS transcriptional regulators, we demonstrated that A20-mediated increase of eNOS was transcriptional and relied on increased expression of the transcription factor Krüppel-like factor (KLF2), and upstream of KLF2, on activation of extracellular signal-regulated kinase 5 (ERK5). Accordingly, ERK5 knockdown or inhibition significantly abrogated A20's ability to increase KLF2 and eNOS expression. In addition, A20 overexpression in HCAEC increased eNOS phosphorylation at Ser-1177, which is key for the function of this enzyme.

**Conclusions:** This is the first report demonstrating that overexpression of A20 in EC increases eNOS transcription in an ERK5/KLF2-dependent manner and promotes eNOS activating phosphorylation. This effect withstands eNOS downregulation by TNF, preventing EC dysfunction in the face of inflammation. This novel function of A20 further qualifies its therapeutic promise to prevent/treat atherosclerosis.

## Introduction

Endothelial nitric oxide (NO) maintains vascular homeostasis and regulates vessel tone (1). Endothelium-derived NO is highly atheroprotective through its combined anti-inflammatory, antithrombotic, antiapoptotic, and antioxidant effects in endothelial cells (EC) (1, 2) and its antiproliferative and proapoptotic effects in intimal smooth muscle cells (SMC) (3). Adequate production of NO by EC relies on constitutively expressed endothelial nitric oxide synthase (eNOS), a calcium/calmodulin-dependent enzyme (4). Regulation of eNOS expression and function in EC is complex, as both physiologic and pathologic stimuli modulate its levels and/or activity. Expression of eNOS, a TATA-less gene, is regulated at the transcriptional, epigenetic, translational, and posttranslational levels (5). At baseline, eNOS expression is maintained by various stimuli, including shear stress, hormones (estrogen), transforming growth factor β, lipoproteins (lysophosphatidylcholine), extracellular ATP, and chronic exercise (6–9). In pathological conditions, eNOS expression decreases in response to inflammatory and metabolic stimuli such as tumor necrosis factor (TNF) or diabetes-associated advanced glycation end-products (5, 10). Multiple transcriptional regulators of eNOS were identified, including Specificity Protein 1 (SP1), SP3, GATA, p53, YY1, AP-1, ETS factors, E74-like ETS transcription factor 1 (ELF1), MYC-associated zinc finger protein (MAZ), Krüppel-like factor (KLF) 2, and KLF4 (11–16). The activity of eNOS is modulated by key post-translational modifications (5, 17), including Ser-1177 and Thr-495 phosphorylation that mark eNOS activation and inhibition, respectively (5, 9, 18–20). In the context of high glucose and diabetes/hyperglycemia, Ser-1177 can also be modified by O-glycosylation, which competes for this residue's phosphorylation to reduce eNOS activity (21).

Decreased eNOS expression and/or activity in response to inflammatory, immune, or metabolic insults is the pathognomonic feature of EC dysfunction and a prime indicator for increased atherosclerotic risk (22). Maintaining adequate eNOS expression and/or activity in the face of atherogenic offenders safeguards vascular homeostasis and prevents or minimizes pathologic vascular remodeling, thereby transforming the natural progression of obstructive vascular disease.

Our group and others have been actively investigating the multiple functions of the potent nuclear factor-kappa B (NF-κB) inhibitory and ubiquitin-modifying protein, A20, also known as Tumor Necrosis Factor Induced Protein 3 (TNFAIP3) (23, 24). The molecular basis for A20's atheroprotective effect has been partially resolved. This pleiotropic protein is anti-inflammatory in EC and SMC through its ability to concomitantly inhibit NF-κB activation in response to inflammatory (TNF), immune (CD40), and oxidative insults, and interrupt atherogenic interferon gamma (IFNγ) signaling (3, 23, 25–27). A20 exerts additional atheroprotective effects in the vessel wall through antiapoptotic and immunomodulatory functions in EC, as well as antiproliferative and proapoptotic functions in SMC, the latter being exclusive to intimal and not medial SMC (3, 28–30). Interestingly, decreased expression of A20 in diabetic patients and mice, as a result of high glucose-induced post-translational O-glycosylation that tags the A20 protein for subsequent ubiquitination and degradation in the proteasome, aggravates and accelerates atherosclerosis in ApoE-null mice rendered diabetic with streptozotocin (31). A similar outcome is noted in A20/TNFAIP3 haplo-insufficient ApoE-null mice (32, 33).

The impact of A20 on eNOS, the molecule at the core of EC health and vascular homeostasis, has not yet been explored. Our study addresses this question by demonstrating that A20 uniquely upregulates basal levels of eNOS through increasing its transcription in an extracellular signal-regulated kinase 5 (ERK5)/KLF2-dependent manner and simultaneously promotes its activating phosphorylation. Importantly, A20 overexpression in EC also significantly limits eNOS transcriptional decline in response to TNF, empowering these cells to resist atherogenic inflammatory insults.

## Methods

### Cell Culture and Reagents

Human coronary artery endothelial cells (HCAEC) derived from six different donors from both genders were purchased from Lonza, Allendale, NJ, and cultured in EGM-2MV BulletKit medium. Bovine aortic endothelial cells (BAEC) were isolated from bovine aortas and cultured as previously described (23). Cells between passages 5–8 were used in the experiments. In select experiments, EC were treated with 200 U/mL TNF for 7 or 24 h (R&D Systems, Minneapolis, MN) and/or 10 μM of the ERK5 inhibitor, XMD8-92 (Cayman Chemicals, Ann Arbor, MI), for 24 h.

### Cell Transduction With Recombinant Adenoviruses

We produced a replication-deficient recombinant adenovirus (rAd) bearing A20 (rAd.A20) (28), using a full-length human A20 expression plasmid (a kind gift of Dr. V. Dixit, Genentech, San Francisco, CA). Control rAd containing β-galactosidase (rAd.βgal) was a gift from Dr. Robert Gerard (University of Texas SW, Dallas, TX). Transgene expression was under the cytomegalovirus promoter. All rAds were purified by the AdenoPure LS Kit (Puresyn, Malvern, PA), and titrated on human kidney embryonic cell line, HEK293 (ATCC, Manassas, VA). HCAEC at 80% confluence were transduced with rAd at a multiplicity of infection (MOI) of 100. Experiments were performed 48 h after transduction. Expression of A20 and β-galactosidase was verified by western blot (WB) analysis, and β-galactosidase expression was also confirmed by X-gal staining. Transduction efficiency reached >90%, without toxicity (28) (Supplementary Figures 1A,B).

In select experiments, HCAEC were first transduced for 24 h with a rAd expressing a human KLF2 silencing short hairpin (Ad-h-KLF2-shRNA) or a control rAd comprising a scramble short shRNA sequence (Ad-GFP-U6-shRNA) at a MOI of 100 (Vector Biolabs, Malvern, PA). Cells were subsequently retransduced with rAd.A20 or rAd.βgal at a MOI of 200–250 or left non-transduced and cultured for an additional 48 h. Both Ad.shRNA constructs used the U6 promoter and expressed enhanced green fluorescent protein (GFP) for the evaluation of transduction efficiency (Supplementary Figure 1C).

### Silencing RNA (siRNA)-Mediated Gene Knockdown

HCAEC were transfected with predesigned human A20 and SP1 silencing RNA probes (siRNA) or AllStars negative control siRNA (Ctrl siRNA), using the HiPerFect Transfection Reagent, per the manufacturer's instructions (Qiagen, Valencia, CA). Experiments were performed 24 h after transfection. Non-transfected cells were also included as controls (Ctrl). The efficiency of gene knockdown was evaluated by quantitative real-time polymerase chain reaction (qPCR) or WB analysis.

### Mice

Male and female C57BL/6 A20 knockout (KO) and heterozygous (HT) mice (a kind gift of Dr. A. Ma, University of California in San Francisco) (34), and wild-type (WT) littermates, were sacrificed at 3–4 weeks of age and their aortae recovered and frozen in liquid nitrogen for RNA extraction and subsequent qPCR analysis. Animals had free access to a standard chow diet and water and were kept in a 12:12-h light–dark cycle. At the time of tissue harvest and euthanasia, mice were anesthetized with isoflurane delivered *via* a precision vaporizer at 5% for induction, followed by 1–3% for maintenance in oxygen. After recovery of the aorta, euthanasia was humanely achieved by exsanguination followed by thoracotomy. All animal experiments were approved by the Institutional Committee for the Use and Care of Laboratory Animals and in accordance with the U.S. Department of Health and Human Services “Guide for the Care and Use of Laboratory Animals.”

### Immunohistochemistry

Brains from 4-week-old A20 HT and KO as well as WT mice were processed for immunohistochemistry (IHC), as previously described (35). In brief, coronal slices were zinc-fixed (BD Pharmigen, San Diego, CA, USA) for 48 h before paraffin embedding and sectioning (6 μm). Sections were deparaffinized, rehydrated, fixed in cold acetone:formalin 95:5 (vol/vol) for 3 min, and then incubated for 1 h with horse serum (7% in PBS) prior to overnight incubation at 4°C with a polyclonal rabbit-anti-eNOS antibody (Abcam Inc., Cambridge, MA, USA). Sections were then treated with H_2_O_2_ 1:100 in PBS for 10 min, incubated with the appropriate secondary IgG antibodies followed by ABC (avidin-biotin complex) reagent (Vector Laboratories, Burlingame, CA, USA) and the ImmPACT 3,3′-diaminobenzidine tetrahydrochloride (DAB) peroxidase substrate (Vector Laboratories, Burlingame, CA, USA).

### Cell Transfection and Reporter Assay

BAEC were grown in six-well-plates until ~70% confluent and transfected with 1.7 μg/well DNA (test plasmids and reporter constructs) using the LipoD293 DNA *in vitro* transfection reagent (SignaGen Laboratories, Rockville, MD), according to the manufacturer's instructions. In all experiments, 0.2 μg of the pRc/Rous sarcoma virus β-galactosidase (RSV.βgal) (Invitrogen, Carlsbad, CA) reporter plasmid and 0.9 μg of the expression plasmid encoding human A20 (pcDNA3.1.A20) or the empty pcDNA3.1 plasmid were co-transfected with 0.6 μg of the luciferase reporter gene (Luc) cloned downstream of a truncation or point mutant human eNOS promoter (a kind gift of Dr. W. Sessa, Yale University School of Medicine) (11). Truncated and mutated eNOS promoter constructs included the following: F1-Luc (full-length eNOS promoter, −1,600 bp), F3-Luc (−1,033 bp), and F3-Luc point mutants at the GATA, p53, SP1, or GATA/SP1 transcription factor binding sites. 48 h after transfection, BAEC were left untreated or treated with 200 U/mL TNF for 7 h. Cells were then harvested using a reporter lysis buffer (Applied Biosystems, Bedford, MA), and extracts were assayed for β-galactosidase and luciferase activities using a Dual-Light System (Applied Biosystems) and Wallac 1420 Victor 2 Microplate Reader (Perkin Elmer, Waltham, MA). Luciferase activity was normalized by β-galactosidase and reported as relative light units (RLU) (23). Data were presented as fold induction relative to the F3-Luc construct.

### Western Blot Analysis

Cells lysates (20–40 μg protein per sample) were separated under reducing conditions by sodium dodecyl sulfate-polyacrylamide gel electrophoresis (Bio-Rad Laboratories, Hercules, CA) and transferred onto a polyvinylidene fluoride membrane (PerkinElmer, Waltham, MA) by semidry electroblotting (36). Membranes were probed with antibodies against human eNOS (BD Bioscience, San Jose, CA; Abcam, Cambridge, MA; Cell Signaling Technology, Danvers, MA) and phospho-eNOS (P-eNOS Ser-1177; BD Bioscience), glyceraldehyde 3-phosphate dehydrogenase (GAPDH), SP1 and β-actin (Santa Cruz Biotechnology, Santa Cruz, CA), A20 (Abcam), and β-galactosidase (Novus Biological, Centennial, CO), followed by the appropriate secondary horseradish peroxidase-conjugated antibodies (Thermo Scientific, Rockford, IL). Protein bands were detected with enhanced chemiluminescence kit (PerkinElmer, Waltham, MA) after exposure to an autoradiography film. The intensity of the scanned bands was quantified by densitometry using the ImageJ 1.41 software (NIH, Bethesda, MD). Alternatively, IRDye® infrared secondary antibodies were used and WB imaging was digitally acquired using the Odyssey® CLx imaging System. The intensity of the bands was quantified using the Image Studio^TM^ Software (Li-COR Inc, Lincoln, NE).

### Quantitative Real-Time Polymerase Chain Reaction

Messenger RNA was isolated using RNeasy Mini kit (Qiagen, Valencia, CA), then cDNA was synthesized using iScript cDNA synthesis kit (Bio-Rad). qPCR was performed using iTaq Universal SYBR Green Supermix (Bio-Rad) and specific primers for human and mouse genes (Supplementary Table 1) (Integrated DNA Technologies, Coralville, IA, and Sigma-Aldrich, St. Louis, MO), using ABI 7500 Fast Real-Time PCR System (Applied Biosystems). Mouse A20 gene expression was quantified using TaqMan Fast Advanced Master Mix and TaqMan Mm00627280_m1 primers (Applied Biosystems). Target gene expression was determined by the relative quantification method using 28S ribosomal RNA or cyclophilin A as housekeeping genes (37).

### Statistical Analysis

Results are reported as mean ± standard error of mean (SEM). Statistical analysis was performed using Prism 8 (GraphPad Software, Inc., La Jolla, CA). Data were analyzed by one- or two-way analysis of variance (ANOVA) followed by Tukey or Bonferroni *post hoc* tests, respectively, or by multiple *t* test comparison. Differences between groups were rated significant at a probability error (*p*) < 0.05.

## Results

### Overexpression of A20 in HCAEC Increases Basal eNOS Expression and Prevents Its Downregulation by TNF

**Figure 1:**
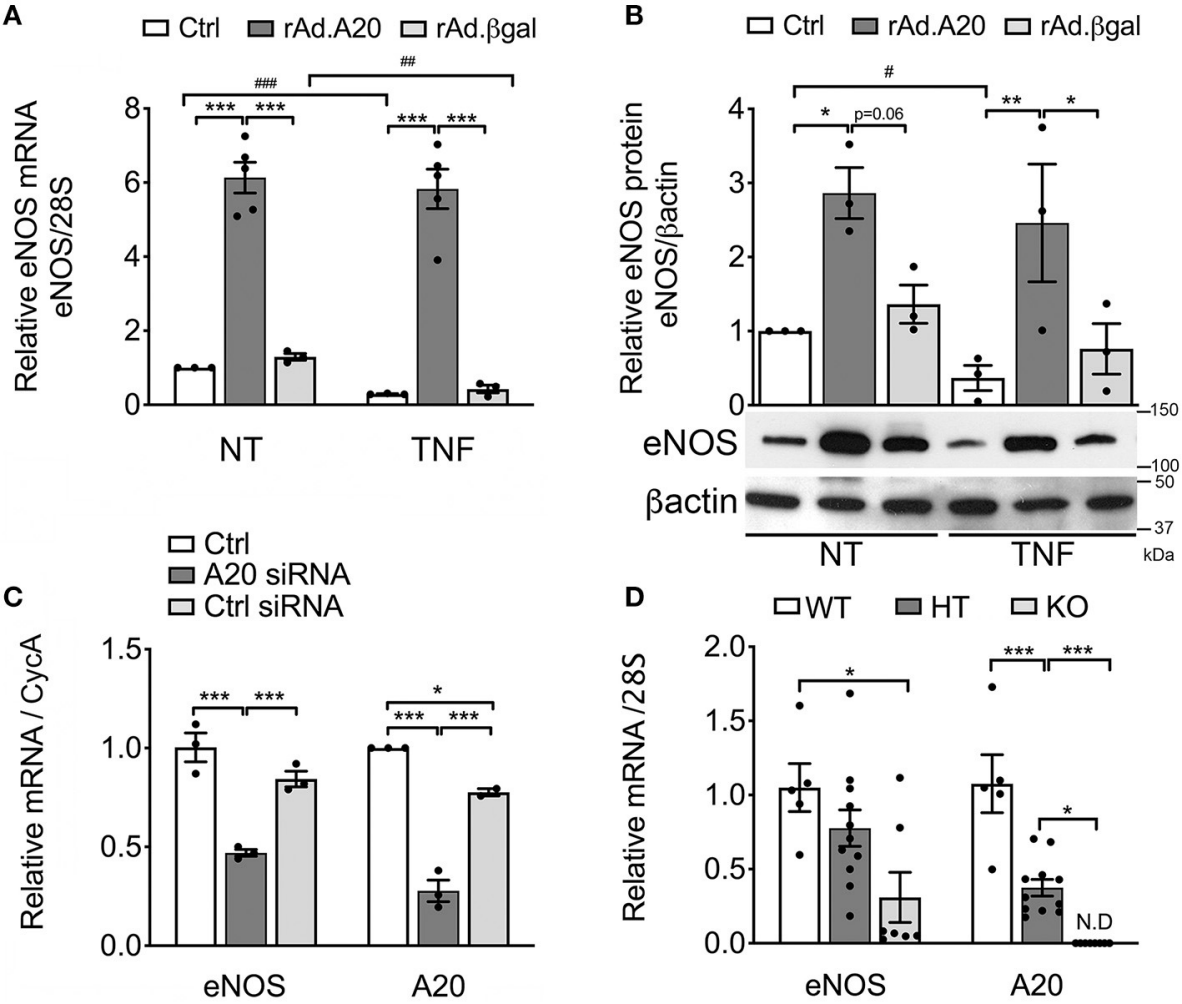
Overexpression of A20 in human coronary artery endothelial cells (HCAEC) increases endothelial nitric oxide synthase (eNOS) expression and prevents its downregulation by TNF, while A20 knockdown decreases eNOS levels. **(A)** Relative eNOS mRNA (qPCR) normalized by mRNA levels of the housekeeping (HKG) gene 28S and expressed as fold change of non-treated (NT) Ctrl HCAEC, and **(B)** eNOS protein (WB) levels in non-transduced HCAEC (Ctrl) and in HCAEC transduced with rAd.A20 or control rAd.βgal (100 MOI), before and 24 h after treatment with TNF. β-Actin was used to correct for loading. **(C)** Relative eNOS and A20 mRNA levels (qPCR) in HCAEC non-transfected (Ctrl) and transfected with A20 siRNA or with AllStars negative control siRNA (Ctrl siRNA) for 24 h, normalized by the mRNA levels of the HKG gene cyclophilin A (CycA), and expressed as fold change of non-transfected Ctrl HCAEC. All data in **(A–C)** are presented as mean ± SEM of three to five independent experiments. Significance between groups was determined by one- or two-way ANOVA followed by the Tukey or Bonferroni multiple comparison *post hoc* test, respectively; *p < 0.05, **p < 0.01, ***p < 0.001. Significance between NT and TNF-treated HCAEC was determined by an unpaired *t* test and depicted as hashtag in lieu of asterisk symbols in order to better differentiate between the two statistical methods used to analyze the data. #*p* < 0.05, ##*p* < 0.01, ###*p* < 0.001. **(D)** Relative eNOS and A20 mRNA levels in the aortae of 3–4-week-old A20 knockout (KO) and heterozygous (HT) mice, as well as wild-type (WT) littermates. Data are presented as mean ± SEM of 5–11 animals/group. Significance between groups was determined by one-way ANOVA followed by the Tukey multiple comparison *post hoc* test; *p < 0.05, **p < 0.01, ***p < 0.001. **(E)** Representative eNOS immunohistochemistry (brown) in the brain of A20 wild-type (WT), heterozygous (HT), and knockout (KO) mice. Photomicrographs are representative of three animals per genotype, magnification = ×400.

To assess the impact of A20 on eNOS expression, we performed gain-of-function studies and checked whether rAd-mediated A20 overexpression in HCAEC affects eNOS mRNA and protein levels. Our results indicate that overexpression of A20 in HCAEC increases basal eNOS mRNA and protein levels by 5- to 6-fold and 3- to 4-fold, respectively, within 48 h after transduction, as compared with non-transduced control (Ctrl) and rAd.βgal-transduced cells (*p* < 0.001 and *p* < 0.05, respectively, Figures 1A,B).

Remarkably, heightened eNOS mRNA and protein levels in A20-overexpressing HCAEC were maintained following TNF treatment. This was in striking contrast with a significant decrease in eNOS mRNA levels in Ctrl (*p* < 0.001) and rAd.βgal-transduced cells (*p* < 0.01, Figure 1A) and with correspondingly lower eNOS protein levels 24 h after TNF treatment (Figure 1B). To our knowledge, these are the first results showing that A20 overexpression in EC significantly increases basal eNOS levels and that this advantage is maintained after TNF treatment.

Additionally, siRNA-mediated A20 knockdown in HCAEC, which resulted in >70% reduction in A20 mRNA levels, decreased basal eNOS mRNA levels by ~50%, as compared with Ctrl and Ctrl siRNA-transfected cells (*p* < 0.05, Figure 1C). These results coincide with *in vivo* data showing that eNOS mRNA levels were significantly lower in the aortae of A20 KO vs. WT littermates (*p* < 0.05, Figure 1D), with levels in the aortae of A20 HT mice fairing in between. They also agree with IHC staining of mouse brain microvasculature, as KO mice showed substantially lower immunostaining for eNOS as compared with WT mice (Figure 1E). Here again, vascular eNOS staining in the brains of HT mice faired in between. Altogether, gain and loss of function studies highlight a novel role for A20 as a physiologic regulator of eNOS expression.

### A20 Increases eNOS Expression by Promoting Its Transcription in a GATA-, SP1-, and p53-Independent Manner

**Figure 2:**
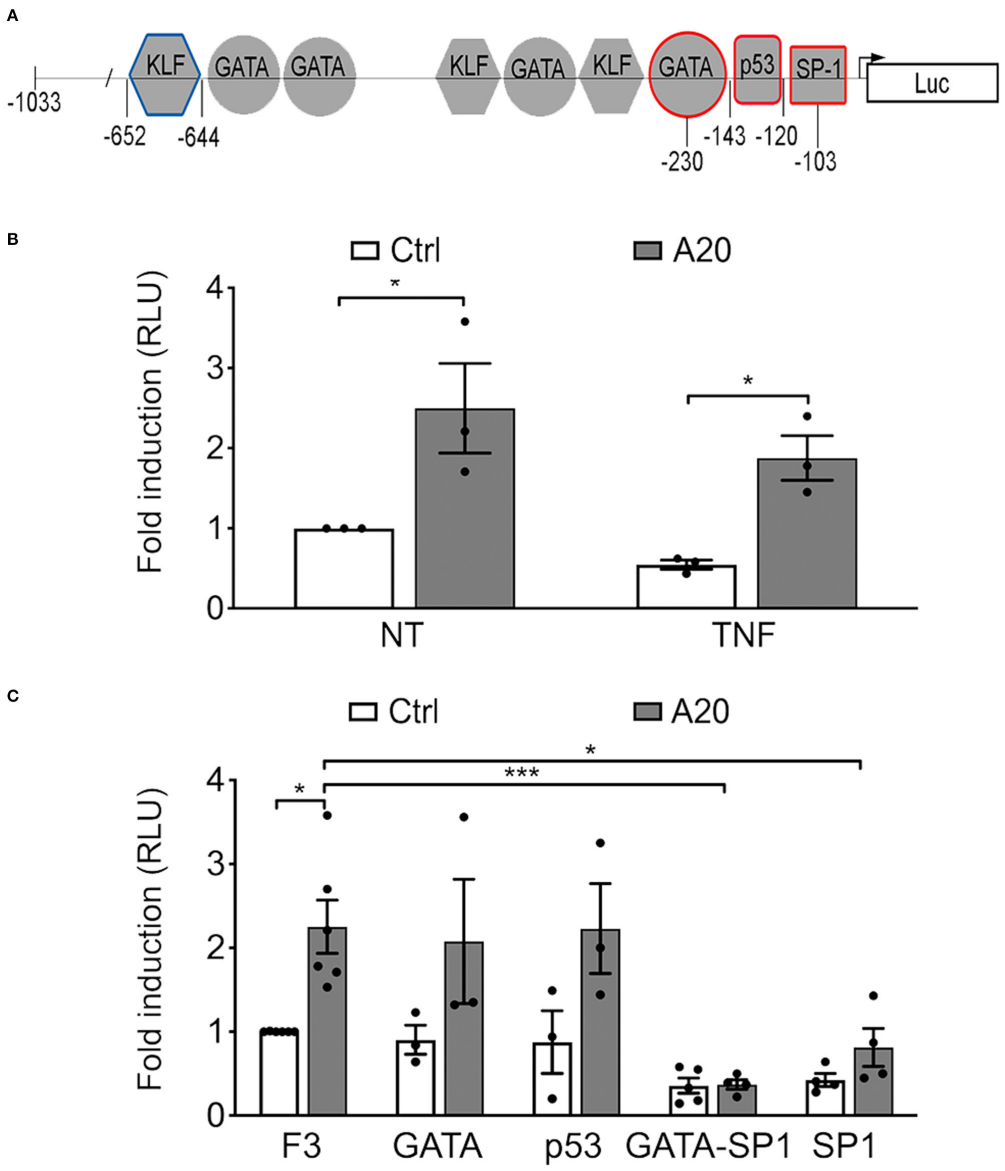
A20-induced eNOS upregulation is transcriptional but independent from GATA, SP1, and p53 binding sites in the eNOS promoter. **(A)** Schematic representation of the truncated F3-Luciferase eNOS promoter construct, encompassing 1,033 bp of the DNA sequence upstream of the transcription start site. Geometrical forms indicate transcription factor binding sites for Krüppel-like factor (KLF), GATA, p53, and SP1, up to position −652. Red markings indicate sites mutated to prevent binding of the respective transcription factor. These mutants were used in experiments shown in **(C)**. The blue marking depicts the prime KLF binding site in the eNOS promoter. **(B,C)** Bovine aortic endothelial cells (BAEC) were co-transfected with various eNOS truncation or point mutant promoter constructs, an RSV-driven βgal reporter, used to correct for transfection efficiency, and either pcDNA3.1-A20 (A20) expression plasmid or an empty pcDNA3.1 vector (Ctrl). Cell lysates were recovered 48 h after transfection and used to measure luciferase and β-galactosidase activities. **(B)** BAEC transfected with the F3-Luc construct were non-treated (NT) or treated with TNF (200 U/mL) for 7 h prior to harvesting. **(C)** BAEC were transfected with F3-Luc eNOS reporter or F3-Luc mutated to preclude GATA, p53, GATA-SP1, or SP1 binding. Luciferase activity, corrected by that of βgal (Luc/βgal), is expressed as relative light units (RLU) and reported as fold change vs. NT F3-Ctrl. Data shown represent mean ± SEM of three to five experiments. *p < 0.05, ***p < 0.001, as determined by two-way ANOVA followed by the Bonferroni *post hoc* test.

To check whether A20-induced upregulation of eNOS expression in EC occurs at the transcriptional level, we transfected BAEC with a plasmid encoding human A20 or an empty control plasmid together with a plasmid comprising the full-length eNOS promoter (F1-Luc) or a minimal 5′ human eNOS promoter fragment, eNOS F3-Luc (−1,033 to +22, Figure 2A), that confers maximal eNOS transcription. Our results confirmed that F3-Luc yielded comparable luciferase levels to F1-Luc (data not shown). BAEC transfected with A20 and the F3-Luc eNOS reporter demonstrated a significant increase in luciferase activity (~2.5-fold) when compared with cells transfected with an empty vector (*p* < 0.05, Figure 2B). This result indicates that A20-mediated upregulation of basal eNOS mRNA levels is transcriptional and occurs within the boundaries of the minimal eNOS promoter. All subsequent experiments were done using the F3-Luc reporter.

Next, we checked whether this effect of A20 on eNOS transcription was affected by TNF treatment. Our results show that heightened luciferase activity in A20-transfected BAEC resists downregulation by treatment with 200 U/mL of TNF for 7 h (*p* < 0.05, Figure 2B).

To elucidate the mechanism(s) by which A20 increases eNOS transcription, we undertook a functional analysis of the eNOS promoter, using the eNOS F3-Luc promoter construct mutated at putative cis-regulatory element binding sites for the inverse GATA element (−231 to −226), p53 (−143 to −120), SP1 (−95 to −109), and GATA/SP1 (−230/−103) (11) (Figure 2A). Our results indicate that mutation of the p53 or the GATA binding sites did not significantly affect baseline eNOS promoter activity in either Ctrl or A20-overexpressing EC, as compared with the intact F3-Luc (Figure 2C). In contrast, luciferase activity of the two eNOS promoter constructs mutated at the SP1 binding sites (SP1 and GATA/SP1 mutants) was substantially lower than that of the intact eNOS F3-Luc reporter (Figure 2C). This agrees with previous reports that SP1 binding to its consensus sequence on the eNOS promoter is indispensable for adequate eNOS transcription (11, 17). However, even when SP1 binding was precluded, the luciferase activity of the GATA/SP1 and the SP1 F3-Luc reporters still trended higher in A20 expressing BAEC (Figure 2C). This implies that A20 overexpression could compensate, at least in part, for the lack of SP1 binding to the eNOS promoter. Because this result does not fully exclude SP1 as one of the potential A20 targets, we also checked whether A20 overexpression in HCAEC affects SP1 protein levels. Reduced SP1 protein levels in the setting of inflammation and diabetes have been previously implicated in decreased eNOS transcription (38). Our results indicate that basal SP1 protein levels in HCAEC are not affected by A20 overexpression (Supplementary Figure 2), which further discounts SP1 as a mediator of the A20 effect on eNOS.

### A20 Increases eNOS Transcription in EC Through an ERK5/KLF2-Dependent Mechanism

**Figure 3:**
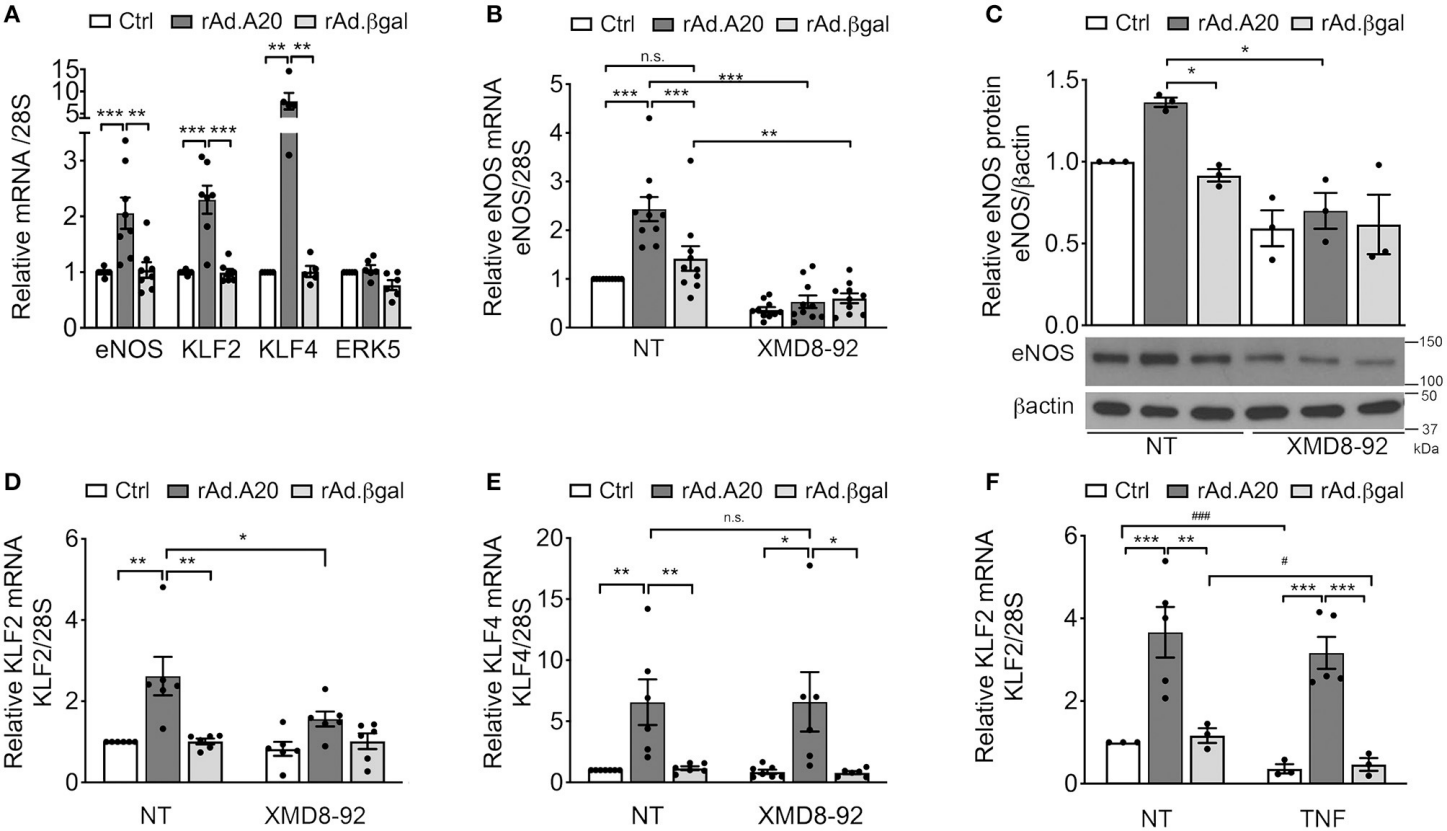
A20 overexpression in HCAEC increases eNOS transcription in an ERK5-dependent manner. **(A)** mRNA levels of eNOS, KLF2, KLF4, and ERK5 were measured by qPCR in non-transduced HCAEC (Ctrl) and HCAEC transduced with rAd.A20 or control rAd.βgal at 100 MOI for 48 h. Data were normalized by the 28S HKG and expressed as mean ± SEM fold change of Ctrl (*n* = 5–9). **(B)** eNOS, **(D)** KLF2, and **(E)** KLF4 mRNA and **(C)** eNOS protein levels were measured by qPCR, and WB in non-transduced HCAEC (Ctrl) and HCAEC transduced with rAd.A20 or control rAd.βgal at 100 MOI for 3 h prior to 24 h treatment with the ERK5 inhibitor, XMD8-92 (10 μM). Graphs in **(B)**, **(D)**, and **(E)** depict relative mRNA levels, normalized by the 28S HKG and expressed as mean ± SEM fold change of non-treated (NT) Ctrl (*n* = 6–10). In **(C)**, β-actin was used to correct for loading. Densitometry results are presented as fold change of NT Ctrl cells (*n* = 3). **(F)** KLF2 mRNA levels were measured by qPCR in non-transduced HCAEC (Ctrl) and in HCAEC transduced with rAd.A20 or control rAd.βgal at 100 MOI for 48 h prior to 24 h treatment with TNF (200 U/mL). Graphs depict relative KLF2 mRNA levels normalized by the 28S HKG and expressed as mean ± SEM fold change of non-treated (NT) Ctrl (*n* = 3–5). *p < 0.05, **p < 0.01, ***p < 0.001, as determined by two-way ANOVA followed by the Bonferroni *post hoc* test. Significance between NT and TNF-treated HCAEC was determined by an unpaired *t* test and depicted as hashtag in lieu of asterisk symbols in order to better differentiate between the two statistical methods used to analyze the data: ^#^*p* < 0.05, ^*###*^*p* < 0.001.

In addition to previously discussed transcriptional regulators of eNOS, two members of the KLF family of zinc finger transcription factors, KLF2 and KLF4, were also identified as central transcriptional regulators of eNOS (13, 16, 39). Several putative binding consensus sequences for KLF were mapped within the eNOS promoter (Figure 2A). To evaluate KLF2 and/or KLF4 involvement in A20-induced upregulation of eNOS transcription, we first checked whether A20 overexpression in HCAEC had any effect on their mRNA levels. Overexpression of A20 in HCAEC significantly increased basal KLF2 (3- to 4-fold, *p* < 0.001) and KLF4 (10-fold, *p* < 0.01) mRNA levels, as compared with Ctrl and rAd.βgal-transduced cells, respectively (Figure 3A). However, A20 overexpression in HCAEC did not affect mRNA levels of ERK5, the transcriptional regulator of KLF2 and KLF4 (Figure 3A). Albeit, we ascertained the implication of the ERK5/KLF2/KLF4 axis in driving A20-mediated upregulation of eNOS transcription by showing that a 24-h incubation of HCAEC with the ERK5-specific inhibitor XMD8-92 abrogated A20-mediated upregulation of eNOS mRNA (*p* < 0.001) and protein (*p* < 0.05) levels (Figures 3B,C). This was also achieved by siRNA-mediated knockdown of ERK5 (70% decrease) in HUVEC and HCAEC (Supplementary Figures 3A,B). Altogether, these results map A20's target at the level of ERK5 activity or upstream of it. Notably, eNOS levels were also moderately reduced, even if not significantly, in NT and rAd.βgal-transduced HCAEC, a stark indicator of the central role ERK5 plays in maintaining basal eNOS expression (40). Pretreatment of HCAEC with XMD8-92 also significantly reduced A20-induced upregulation of KLF2 (*p* < 0.05) but not KLF4 mRNA levels (Figures 3D,E). This result identifies KLF2 as the dominant KLF member driving A20-induced eNOS expression in EC. Akin to eNOS, A20-mediated upregulation of KLF2 mRNA levels was preserved following TNF treatment (200 U/mL, 24 h), while those levels decreased by >50% in Ctrl (*p* < 0.001) and rAd.βgal-treated (*p* < 0.05) HCAEC (Figure 3F).

**Figure 4:**
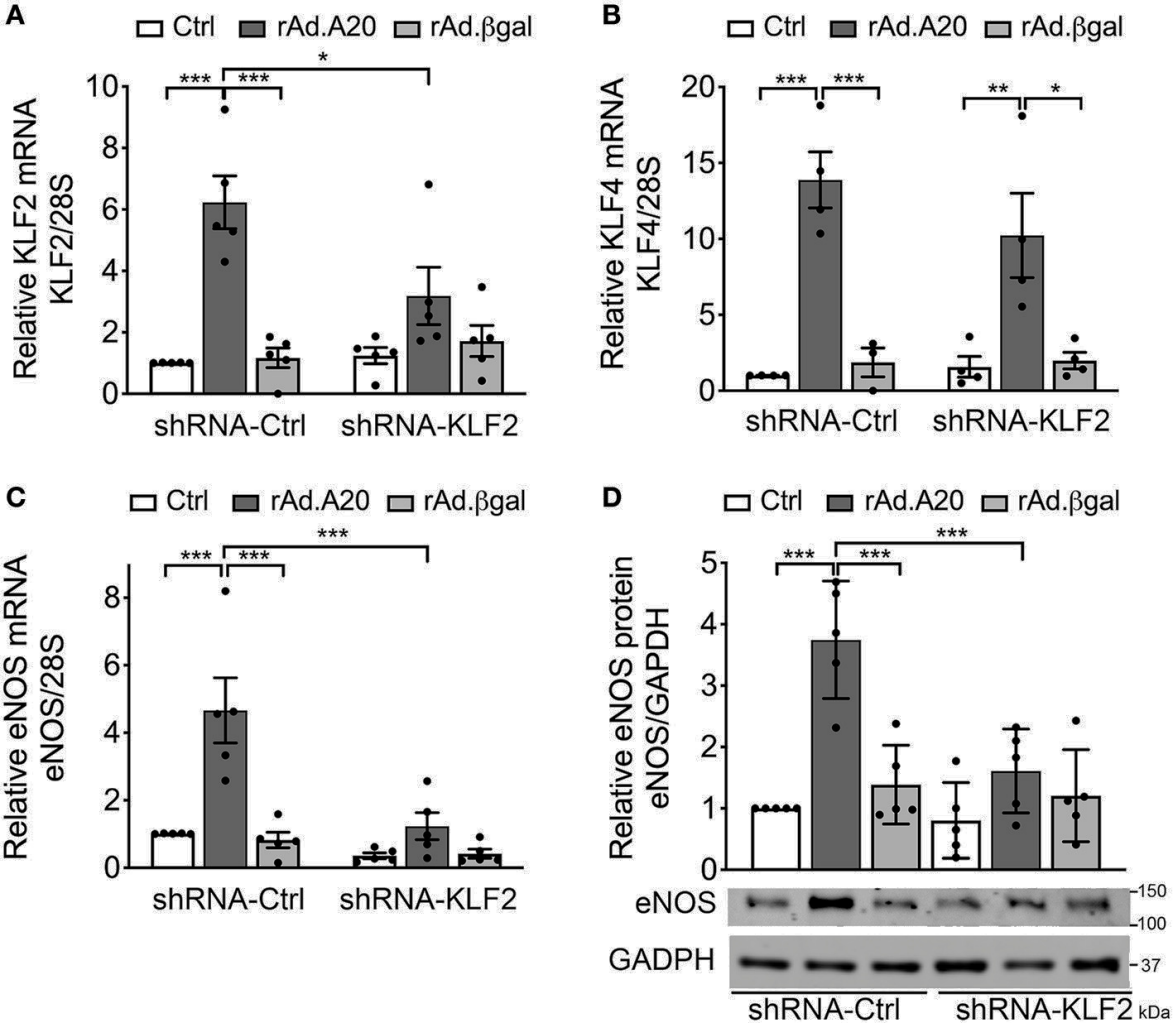
A20 overexpression in HCAEC increases eNOS transcription in a KLF2-dependent manner. HCAEC were transduced with 100 MOI of shRNA-KLF2 or scramble shRNA-Ctrl for 24 h, then retransduced with rAd.A20 or control rAd.βgal at 200–250 MOI for 48 h or left non-transduced (Ctrl). Cell lysates were evaluated by qPCR for mRNA levels of **(A)** KLF2, **(B)** KLF4, and **(C)** eNOS. Graphs shown depict relative mRNA levels, normalized by the 28S HKG and expressed as mean ± SEM fold change of Ctrl shRNA-Ctrl cells (*n* = 3–6) and by **(D)** WB for eNOS protein expression. GAPDH was used to correct for loading. Densitometry results are presented as fold change of Ctrl shRNA-Ctrl cells and expressed as mean ± SEM (*n* = 4). *p < 0.05, **p < 0.01, ***p < 0.001, as determined by two-way ANOVA followed by the Bonferroni *post hoc* test.

To further confirm the role of KLF2 as the prime mediator of A20-induced upregulation of eNOS transcription, we knocked down KLF2 using a rAd-expressing shRNA. HCAEC were first transduced with 100 MOI of shRNA-KLF2 or a shRNA control (shRNA-Ctrl), and then again 24 h later with 200–250 MOI of either rAd.A20 or rAd.βgal. Dual transduction led to shRNA and transgene expression in >95% of the cells without toxicity (Supplememtary Figures 1B,C). HCAEC transduced with both shRNA-Ctrl and rAd.A20 had significantly higher KLF2 (6-fold, *p* < 0.001), KLF4 (12-fold, *p* < 0.001), and eNOS (5-fold, *p* < 0.001) mRNA levels, as well as eNOS protein levels (3-fold, *p* < 0.01),compared with HCAEC transduced with shRNA-Ctrl only or with shRNA-Ctrl and rAd.βgal (Figures 4A–D). These results confirm that this dual transduction protocol does not affect A20's ability to increase KLF2, KLF4, and eNOS expression levels. Transduction of HCAEC with shRNA-KLF2 reduced KLF2s mRNA levels by 50%, but did not affect KLF4 mRNA levels in A20-overexpressing cells, confirming the specificity of the shRNA KLF2 (Figures 4A,B). Importantly, KLF2 knockdown significantly decreased A20-induced upregulation of eNOS mRNA (*p* < 0.001) and protein (*p* < 0.01) levels, which confirmed that A20-induced upregulation of eNOS transcription and expression was KLF-2 dependent (Figures 4C,D). Taken altogether, our data uncover a novel function of A20 in EC as a positive regulator of eNOS expression through an ERK5/KLF2-dependent manner.

**Figure 5:**
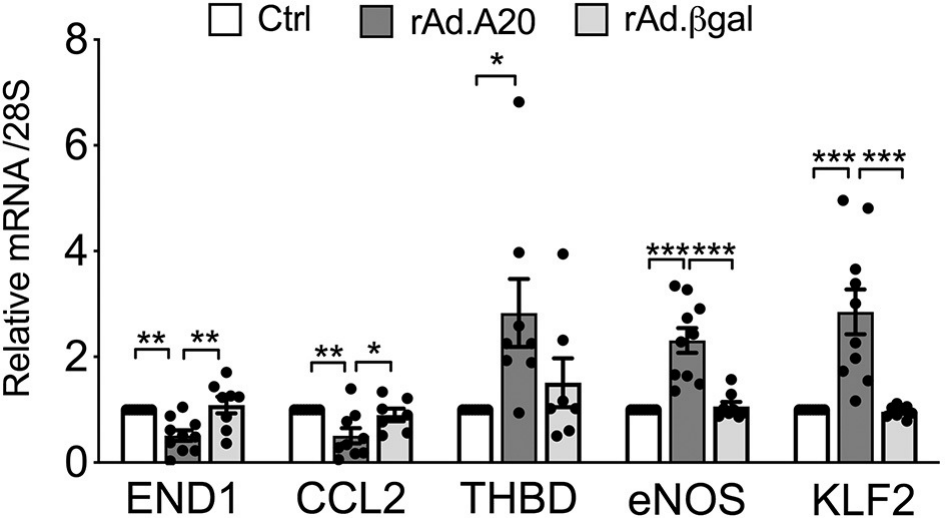
Overexpression of A20 in HCAEC modulates the expression levels of KLF2-dependent genes to increase EC homeostasis. mRNA levels of endothelin-1 (END1), chemokine C-C motif ligand 2 (CCL2)/monocyte chemoattractant protein-1 (MCP1), thrombomodulin (THBD), eNOS, KLF2, and A20 were measured by qPCR in non-transduced HCAEC (Ctrl) and HCAEC transduced with rAd.A20 or control rAd.βgal. Data were normalized by the 28S HKG and expressed as mean ± SEM fold change of Ctrl (*n* = 8–10). *p < 0.05, **p < 0.01, ***p <0.001, as determined by one-way ANOVA followed by Tukey *post hoc* test.

In addition to eNOS, other key transcriptional targets of KLF2 were modulated (41), as evidenced by a significant increase in basal mRNA levels of thrombomodulin and a significant decrease in mRNA levels of endothelin-1 and CCL-2—also known as monocyte chemoattractant protein 1 (MCP1)—in rAd.A20 vs. Ctrl and rAd.βgal-transduced HCAEC (Figure 5).

### Overexpression of A20 Increases the Activating Ser-1777 Phosphorylation of eNOS

**Figure 6:**
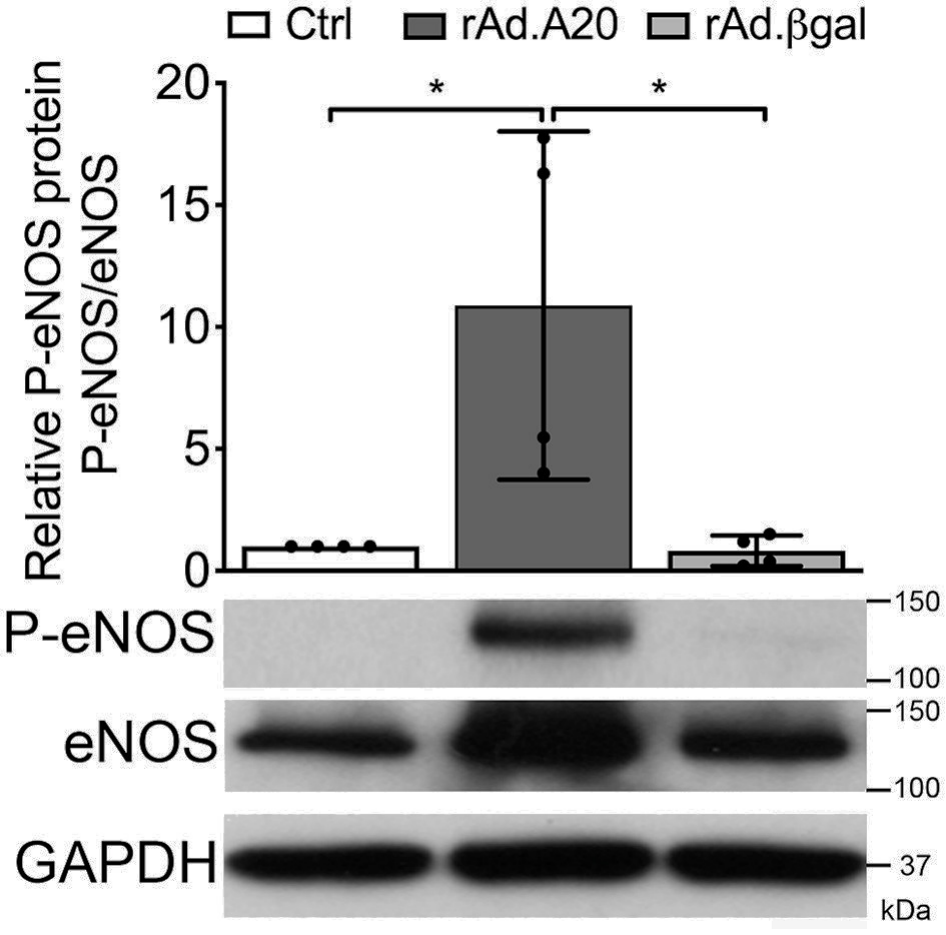
A20 overexpression increases basal Ser-1177 eNOS phosphorylation in HCAEC. Representative WB of total eNOS and P-eNOS (Ser-1177) in non-transduced HCAEC (Ctrl) and HCAEC transduced with rAd.A20 or rAd.βgal at 100 MOI for 48 h; GAPDH was used to correct for loading. Densitometry results are presented as mean fold change of Ctrl ± SEM (*n* = 4). *p < 0.05, as determined by one-way ANOVA followed by the Tukey *post hoc* test.

In addition to increasing eNOS levels, A20 overexpression significantly promoted basal phosphorylation of eNOS at Ser-1177, which is key for eNOS activation (42). At quiescence, eNOS phosphorylation was low in Ctrl and rAd.βgal-transduced HCAEC, whereas it was well-evident in A20-overexpressing cells (Figure 6). By calculating the ratio of phosphorylated over total eNOS protein, we verified that increased basal eNOS phosphorylation in A20-transduced cells did not merely reflect higher protein levels but truly its increased phosphorylation (*p* < 0.05, Figure 6). This result further qualifies the positive effect of A20 on eNOS, not only by increasing its transcription but also enhancing its activating phosphorylation.

## Discussion

Decreased bioavailability of eNOS-derived vascular NO is the prime characteristic of EC dysfunction that preludes the development of vascular pathologies (1, 2). The importance of eNOS in the hierarchy of physiologic atheroprotective mechanisms is supported by heightened risk for atherosclerosis, myocardial infection, heart failure, and hypertension in eNOS KO mice (43, 44). Expression and/or activity of eNOS substantially decreases in response to classic atherogenic culprits such as chronic inflammation (TNF, interleukin 1, and IFNγ), oxidative stress, hypoxia, hyperglycemia, and dyslipidemias (45–48). Numerous mechanisms account for such reduction, ranging from deficient eNOS dimerization due to reduced levels of the cofactor tetrahydrobiopterin, altered coupling, decreased mRNA half-life, inactivating posttranslational modifications such as competitive inhibitory O-glycosylation of Ser-1177 that precludes its activating phosphorylation, and reduced transcription through negative effects on one or more of eNOS transcriptional activators (5, 17, 21, 49).

Substantial efforts have been deployed to identify novel strategies to maintain or restore eNOS expression and/or function and uphold vascular health to reduce risk of cardiovascular disease. Our data demonstrate for the first time that overexpression of the potent NF-κB inhibitory and ubiquitin-editing protein A20 in EC fulfills this goal by significantly increasing basal eNOS mRNA and protein levels. Remarkably, this advantage resists TNF-induced downregulation of eNOS expression. Heightened levels of eNOS mRNA (6-fold) and protein (3-fold) were still maintained in A20-overexpressing HCAEC despite TNF treatment. This novel function of A20 adds an important dimension to the other atheroprotective attributes of this versatile molecule. This effect of A20 was not limited to HCAEC but was also evident in other vascular beds such as HUVEC (Supplementary Figure 3).

Conversely, A20 knockdown reduced basal eNOS expression by >50% in HCAEC. This result underscores the physiologic role A20 plays to maintain optimal eNOS expression in support of endothelial health. It also maps a novel molecular link between decreased A20 levels, such as in subjects harboring specific TNFAIP3 single nucleotide polymorphisms or in poorly controlled diabetics where O-glycosylation of the A20 protein leads to its degradation and increased incidence of EC dysfunction and atherogenesis (31, 50). Our data showing decreased expression of eNOS in the vasculature of both A20 homozygous and heterozygous KO mice lend additional support to this statement, especially in light of A20 haploinsufficiency accelerating and aggravating atherosclerotic lesions in atheroprone ApoE-null mice (32).

The impact of A20 gain or loss on overall eNOS expression corresponded with increased or decreased mRNA levels of this enzyme, suggesting an effect on either eNOS mRNA stability/half-life or transcription (46). Using a classic assay that evaluates mRNA half-life after transcription is inhibited by actinomycin D, we ruled out any effect of A20 on eNOS mRNA stability (data not shown). Rather, using eNOS reporter assays, we showed that A20-mediated upregulation of eNOS mRNA was transcriptional and that this advantage was maintained following TNF treatment.

As previously noted, eNOS transcription is regulated by the complex cooperation of a number of transcription factors, including SP1, AP-1, p53, GATA, KLF2, and KLF4 (11–17, 41). Our data using a series of eNOS promoters mutated at critical binding sites for GATA, p53, AP-1, and SP1 ruled out the implication of any of them in A20-mediated upregulation of eNOS. Specifically, mutation of the inverse GATA element, p53, or AP1 binding site did not affect basal or A20-induced upregulation of the eNOS reporter activity. On the other hand, mutation of the SP1 *cis*-element binding site (−109 to −95) significantly decreased basal eNOS reporter luciferase activity in both A20-transfected and control EC. This result aligns with a number of previous publications documenting the indispensable role of SP1 binding at this proximal eNOS promoter site in maintaining steady-state eNOS transcription (11, 12, 17). However, even when SP1 binding was precluded, overexpression of A20 still increased by ~2-fold eNOS reporter activity, bringing it up to baseline levels of Ctrl. This remarkable result not only indicates that the effect of A20 is independent from SP1, but also that A20 can, at least in part, compensate for the loss of this key driver of eNOS transcription. Although A20-induced upregulation of basal eNOS transcription is SP1 independent, one still expects that overexpression of A20 in EC may prevent TNF-induced downregulation of eNOS transcription by preventing NF-κB activation-induced exclusion of SP1 from the eNOS promoter (17).

KLF2 and KLF4 are two other key transcriptional regulators of eNOS (13, 16, 41, 51, 52). Several KLF consensus binding sites were identified in the human eNOS promoter, including at position −652 to −644 that is deemed essential for KLF-mediated transactivation of the eNOS promoter (13). Interestingly, our data show that A20 overexpression in HCAEC significantly increases KLF2 and KLF4 mRNA levels, in a way that is commensurate to the levels achieved when EC are cultured under laminar flow (15, 39, 53). This suggests that overexpression of A20 can compensate for the absence of an atheroprotective laminar pulsatile shear stress, thereby reducing atherogenic risk in vascular beds and regions that are impeded by perturbed flow (54–56).

Similar to eNOS, KLF2 and/or KLF4 transcription is induced by atheroprotective laminar flow/pulsatile shear stress and decreased by atherogenic disturbed flow/oscillatory shear (39, 55, 57). Identifying KLF2 and/or KLF4 as the likely link(s) between A20 and heightened eNOS transcription broadens the list of atheroprotective and anti-inflammatory targets that A20 impacts in EC. In addition to increasing eNOS transcription, KLF2, and to some extent KLF4, safeguards EC barrier function, prevents vascular inflammation and thrombosis by increasing expression of many other atheroprotective genes while repressing that of atherogenic ones, and modulates monocyte's inflammatory responses (13, 16, 54, 58–61). Our data showing that overexpression of A20 in HCAEC significantly increases basal expression of the antithrombotic molecule thrombomodulin, while significantly decreasing that of atherogenic endothelin-1 and proinflammatory/chemotactic CCL-2—also known as MCP1—downstream of KLF2 (41), support this claim (Figure 5). The potent atheroprotective properties of KLF2 are highlighted by the observation that mere KLF2 haploinsufficiency, akin to A20, accelerates and aggravates atherosclerotic lesions in ApoE-null mice (32, 33, 62). KLF2 expression is characteristically high in atherosclerosis-resistant regions of the vasculature but nearly absent at branch points and other atheroprone vascular segments with evidence of inflammation and NF-κB activation (39, 54, 63). Atheroprone flow, proinflammatory cytokines, and hyperglycemia/diabetes substantially decrease KFL2 expression (39, 54, 63, 64). We confirmed that KLF2 mRNA levels in HCAEC, akin to eNOS, decrease following TNF treatment and that A20 overexpression remarkably prevents this decline.

**Figure 7:**
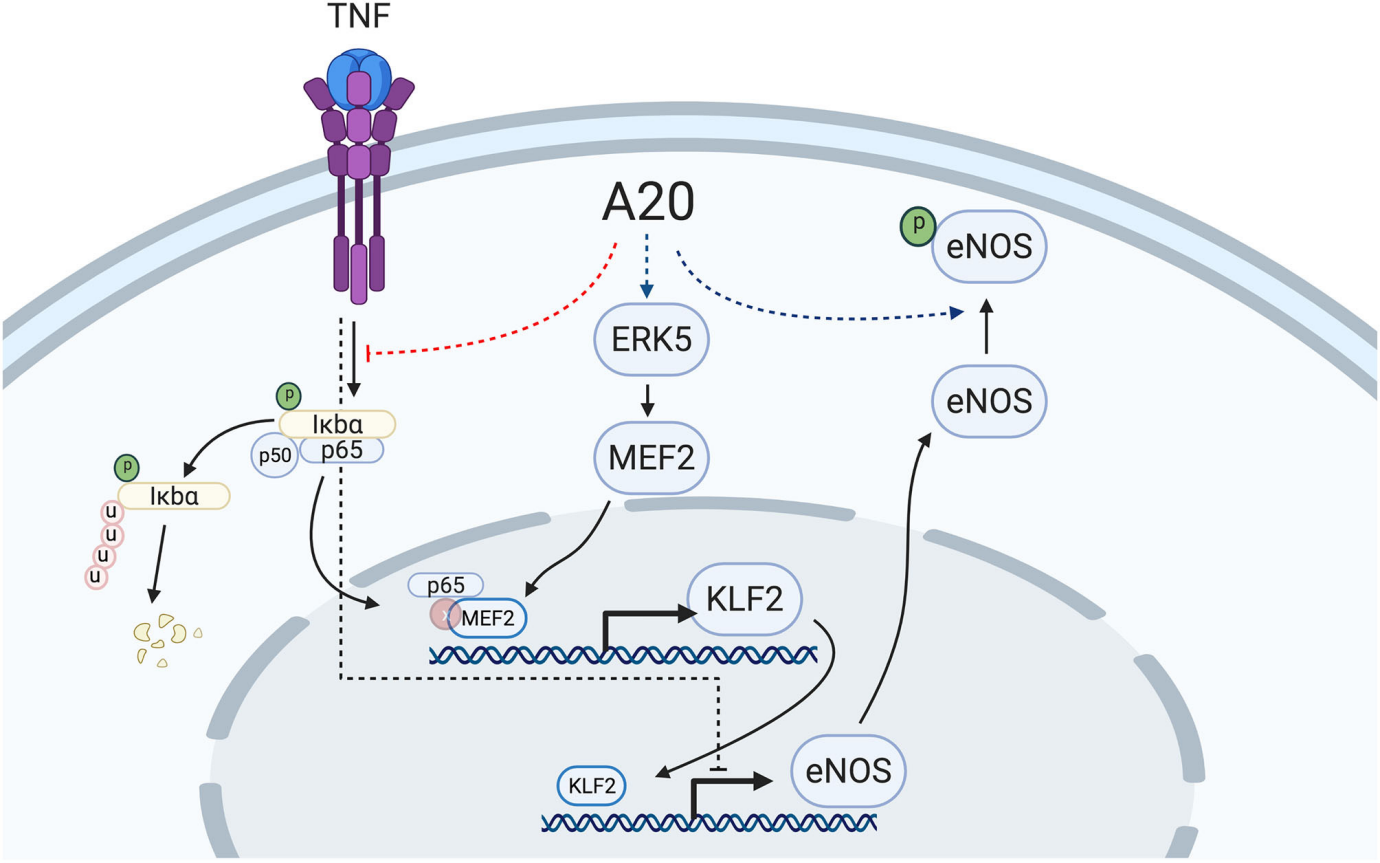
Proposed mechanism of A20-induced prevention of EC dysfunction by increasing eNOS transcription and Ser-1177 activating phosphorylation. A20 overexpression in EC increases basal eNOS transcription in an ERK5-dependent manner with subsequent upregulation of the eNOS transcriptional regulator, KLF2. Overexpression of A20 in EC also precludes TNF-induced decrease of eNOS and KLF2, likely through inhibition of NF-κB activation, precluding p65 translocation to the nucleus and its subsequent binding to the KLF2 transcriptional regulator, MEF2. Additionally, A20 overexpression in EC increases basal Ser-1177 phosphorylation of eNOS, which is indispensable for the function of this enzyme. The mechanism(s) behind this latter effect remains to be elucidated. Red dotted lines delineate downregulation by A20, while blue dotted lines delineate upregulation by A20. Scheme was created using BioRender.com.

In the vascular endothelium, KLF2 is transcriptionally regulated in a MEK5/ERK5/MEF2-dependent manner (55, 60, 65, 66). ERK5, also known as big mitogen-activated protein kinase (BMK1), is mostly activated through post-translational modifications in response to shear stress, growth factors, and cytokines (66–69). In turn, ERK5 activates MEF2 (myocyte enhancer factor 2), which promotes its binding to the KLF2 promoter to drive its transcription (65). Numerous vascular anti-inflammatory functions have been attributed to the MEK5/ERK5/MEF2 pathway, to which KLF2 greatly contributes (13, 66, 68). A20 overexpression did not affect ERK5 expression levels. However, the critical implication of this kinase in driving A20-induced upregulation of eNOS transcription was still evidenced by our data showing that siRNA-mediated knockdown of ERK5 or inhibition of ERK5 activity by XMD8-92 blunted A20-induced upregulation of eNOS and of its upstream transcriptional regulator KLF2, but interestingly not KLF4. This latter result indicates that increased eNOS transcription in A20-overexpressing EC is mostly KLF2 dependent. It also implies that increased KLF4 expression in A20-overexpressing HCAEC is not only ERK5 independent but also cannot compensate for KLF2 in driving the A20-mediated increase in eNOS transcription. This result differs from previous reports of KLF4 as a driver of eNOS transcription (52), a discrepancy that may result from differences in EC beds that were used, i.e., dermal microvascular EC vs. HCAEC, which we believe to be more relevant to EC dysfunction. Other evidence supports the dominant role of KLF2 in promoting eNOS transcription, including our data showing that a mere 50% reduction in A20-induced KLF2 upregulation in HCAEC is sufficient to annul A20-mediated increase in eNOS, and that KLF2 expression levels decrease in parallel to eNOS levels when EC are treated with TNF (Figure 3F) (13, 17, 39). This contrasts with KLF4 mRNA levels that are either unchanged or even increased after TNF treatment (15) (Supplementary Figure 5). The mechanism by which TNF decreases KLF2 transcription results from the binding of MEF2 to the p65 subunit of NF-κB at the level of the KLF2 promoter (65). A20 is a potent inhibitor of NF-κB activation that precludes nuclear translocation of p65 (70), and this mechanism could contribute to A20 overexpression maintaining KLF2 and eNOS expression following exposure to TNF (Figure 7). We verified in experiments using TNF that overexpression of A20 in HCAEC significantly inhibited TNF-mediated transcription of the bona fide NF-κB target genes VCAM-1 and ICAM-1 (Supplementary Figure 5).

Because the atheroprotective effect of eNOS relies on its enzymatic activity, we checked whether overexpression of A20 in HCAEC affected eNOS phosphorylation of this enzyme activating site at Ser-1177. Phosphorylation of this residue is classically associated with enhanced NO production, even if recent reports suggest that it is not strictly necessary for eNOS activity (42, 71). Our data indicate that overexpression of A20 in HCAEC did not impair EC's ability to phosphorylate eNOS at Ser-1177 in response to activators such as TNF or vascular endothelial growth factor (data not shown) and even promoted this phosphorylation at baseline. This observation agrees with previous data by Li *et al*. showing that A20 knockdown in HUVEC reduces eNOS activating phosphorylation in a TAK1/p38 MAPK-dependent manner (72). Conversely, A20 overexpression inactivates TAK1 and suppresses p38 MAPK to restore eNOS phosphorylation (72). Future work is planned to investigate whether the TAK1/p38 pathway is relevant to our data in HCAEC and also to evaluate the alternative contribution of other molecular mechanisms. We already ruled out the contribution of ERK5 activation in A20-mediated increase of eNOS phosphorylation, as this effect was not abrogated when EC were preincubated with XMD8-92 (Supplementary Figure 6A). We also excluded any impact A20 might have on eNOS dimerization as a contributor to increased eNOS phosphorylation. Indeed, A20 overexpression in EC did not modify the ratio of eNOS dimers over monomers (Supplementary Figure 6B).

In summary, data presented in this manuscript uncover a novel function of A20 as a key physiologic regulator of EC homeostasis through its ability to enhance eNOS transcription in an ERK5/KLF2-dependent manner while simultaneously promoting eNOS activating phosphorylation. Remarkably, this effect of A20 resists inflammation-mediated downregulation of eNOS and its upstream atheroprotective transcriptional KLF2. This novel attribute of A20 further qualifies its broad atheroprotective potential and justifies our continued pursuit of A20-based therapies to prevent/treat atherosclerotic vascular disease. From a translational standpoint, A20-mediated upregulation of KLF2/eNOS, which remains contained within the boundaries of physiological levels induced by shear stress, is likely more advantageous than direct overexpression of either molecules, as supraphysiologic levels of KLF2 or eNOS may result in undesirable side effects.

## Data Availability Statement

The raw data supporting the conclusions of this article will be made available by the authors, without undue reservation.

## Ethics Statement

The animal study was reviewed and approved by Institutional Committee for Use and Care of Laboratory Animals at the Beth Israel Deaconess Medical Center. Written informed consent was obtained from the owners for the participation of their animals in this study.

## Author Contributions

CA, EK, SE, SD, LC, BT, GS, AL, FK, MA, and HM performed experiments. CA, EK, SE, and CF analyzed data and designed the study. CA, EK, and CF wrote the manuscript. All authors contributed to the article and approved it for publication.

## Conflict of Interest

The authors declare that the research was conducted in the absence of any commercial or financial relationships that could be construed as a potential conflict of interest.

## Abbreviations

βgal: β-galactosidase
BAEC: bovine aortic endothelial cells
EC: endothelial cells
eNOS: endothelial nitric oxide synthase
ERK5: extracellular signal-regulated kinase 5
GAPDH: glyceraldehyde 3-phosphate dehydrogenase
HCAEC: human coronary artery endothelial cells
HT: heterozygous
HUVEC: human umbilical vein endothelial cells
IHC: immunohistochemistry
IFNγ: interferon gamma
GFP: green fluorescent protein
KLF: Krüppel-like factor
KO: knockout
MAPK: mitogen-activated protein kinase
MEF2: myocyte enhancer factor 2
MOI: multiplicity of infection
NF-κB: nuclear factor-kappa B
NO: nitric oxide
P-eNOS: phosphorylated-eNOS
qPCR: quantitative real-time polymerase chain reaction
rAd: recombinant adenovirus
RSV: pRc/Rous sarcoma virus
RLU: relative light units
shRNA: short hairpin RNA
siRNA: silencing RNA
SMC: smooth muscle cells
SP1: specificity protein 1
TNF: tumor necrosis factor
TNFAIP3: tumor necrosis factor induced protein 3
WB: western blot
WT: wild type.
