# Supplementary material for: A20/TNFAIP3 Increases ENOS Expression in an ERK5/KLF2-Dependent Manner to Support Endothelial Cell Health in the Face of Inflammation
Source: Front Cardiovasc Med. 2021 May 7;8:651230. doi: 10.3389/fcvm.2021.651230 (PMC8138474; doi:10.3389/fcvm.2021.651230)
Supplement: Supplementary file 1 [file Data_Sheet_1.docx]

**Supplemental Material**

**Supplemental Methods**

**Cell culture**

Human umbilical vein endothelial cells (HUVEC) (Lonza, Allendale, NJ) were cultured in EGM-MV Bullet Kit medium (Lonza), at 37°C in a 5% CO_2_ humidified air incubator. Confluent cells between passages 4 to 6 were used in all experiments.

**Analysis of eNOS dimers and monomers by low-temperature SDS-PAGE western blot**

HCAEC non-transduced (NT) or transduced with rAd.A20 or rAd.βgal at 100 MOI for 48 h were harvested with Radioimmunoprecipitation assay (RIPA) lysis buffer containing protease inhibitors (Roche, Indianapolis, NJ) and phosphatase inhibitors (Boston Bioproducts, Ashland, MA). Protein lysates (35-45 μg) were mixed with Laemmli sample buffer without β-mercaptoethanol (non-reducing conditions). Samples were kept on ice at all times to preserve the integrity of the eNOS dimers. Samples processed under standard reducing (2.5% β-mercaptoethanol) and denaturing (5 minutes boiling) conditions were run in parallel. Monomer and dimer forms of eNOS were separated on prechilled 7.5% polyacrylamide gels and running buffer (Bio-Rad, Hercules, CA), in an ice bath in the cold room for 8 h at 40 volts. Proteins were transferred to a polyvinylidene fluoride (PVDF) membranes by semidry electroblotting, then probed with antibodies against human eNOS and glyceraldehyde 3-phosphate dehydrogenase (GAPDH) (Santa Cruz Biotechnology, Santa Cruz, CA), followed by the appropriate IRDye^®^ infrared secondary antibodies. WB imaging was digitally acquired using the Odyssey^®^ CLx Imaging System. Intensity of the bands was quantified using the Image Studio^TM^ Software (Li-COR Inc, Lincoln, NE).

**Silencing RNA (siRNA)-mediated ERK5 knockdown**

HUVEC and HCAEC were transfected with predesigned human ERK5 silencing RNA probes (siRNA) or AllStars Negative Control siRNA (Ctrl siRNA) (Qiagen, Valencia, CA), using the HiPerFect Transfection Reagent, as per the manufacturer’s instructions (Qiagen). Four h after transfection, cells were transduced with rAd.A20 or control rAd.βgal at 100 MOI for an additional 24 h prior to mRNA extraction. Non-transfected cells were also included as controls (Ctrl). Efficiency of gene knockdown was evaluated by quantitative real-time Polymerase Chain Reaction (qPCR). mRNA was isolated with RNeasy Mini Kit (Qiagen, Valencia, CA) and used for generation of cDNA using iScript cDNA Synthesis Kit (Bio-Rad, Hercules, CA). qPCR was performed using iTaq Fast SYBR Green Supermix with ROX (Bio-Rad, Hercules, CA) and gene specific primers (Integrated DNA Technologies, Coralville, IA and Sigma-Aldrich, St. Louis, MO) using ABI 7500 Fast Real-Time PCR System (Applied Biosystems, Inc., Foster City, CA). Target gene expression was determined by the relative quantification method using 28S ribosomal RNA as housekeeping gene[^34^](#_ENREF_34).

**Supplementary Figures**

**
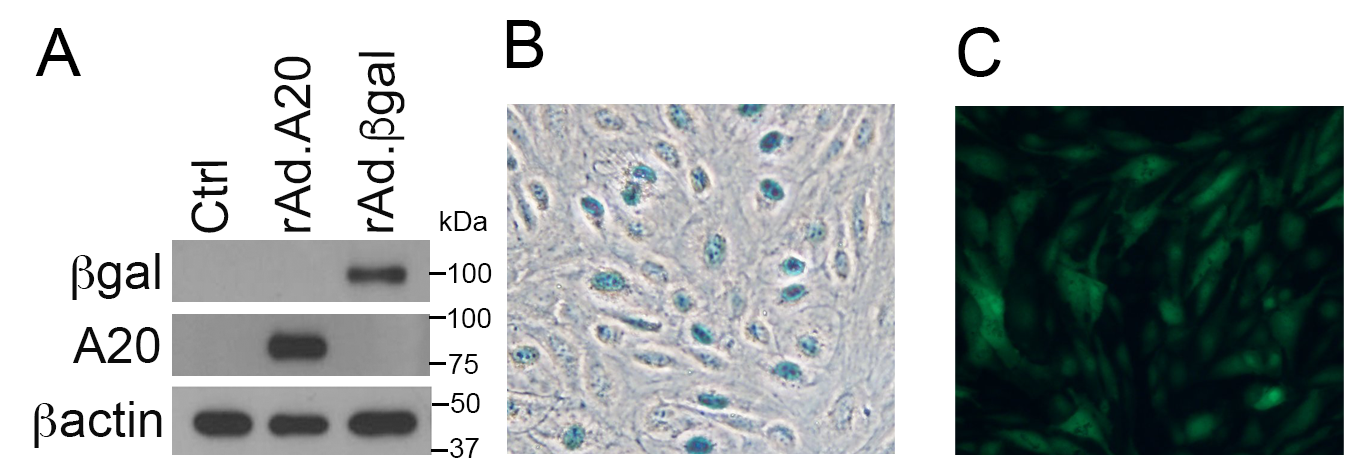
**

**Supplementary Figure 1. rAd-mediated gene transfer yields robust transgene expression in >90% of HCAEC without causing toxicity.** **A.** Non-transduced HCAEC (Ctrl) and HCAEC transduced with rAd.A20 or control rAd.βgal at 100 MOI for 48 h were harvested for protein extraction and analyzed by western blot for expression of A20 and βgal. βactin was used as a loading control. Representative images of **B.** light microscopy, and **C.** fluorescence microscopy show nuclear X-gal staining of βgal activity (blue) and cytoplasmic GFP expression (green) of scramble shRNA (shRNA-Ctrl) containing the reporter gene GFP in HCAEC transduced with 100 MOI of shRNA-Ctrl and 24 h later transduced with 200 MOI of rAd.βgal for additional 48 h.


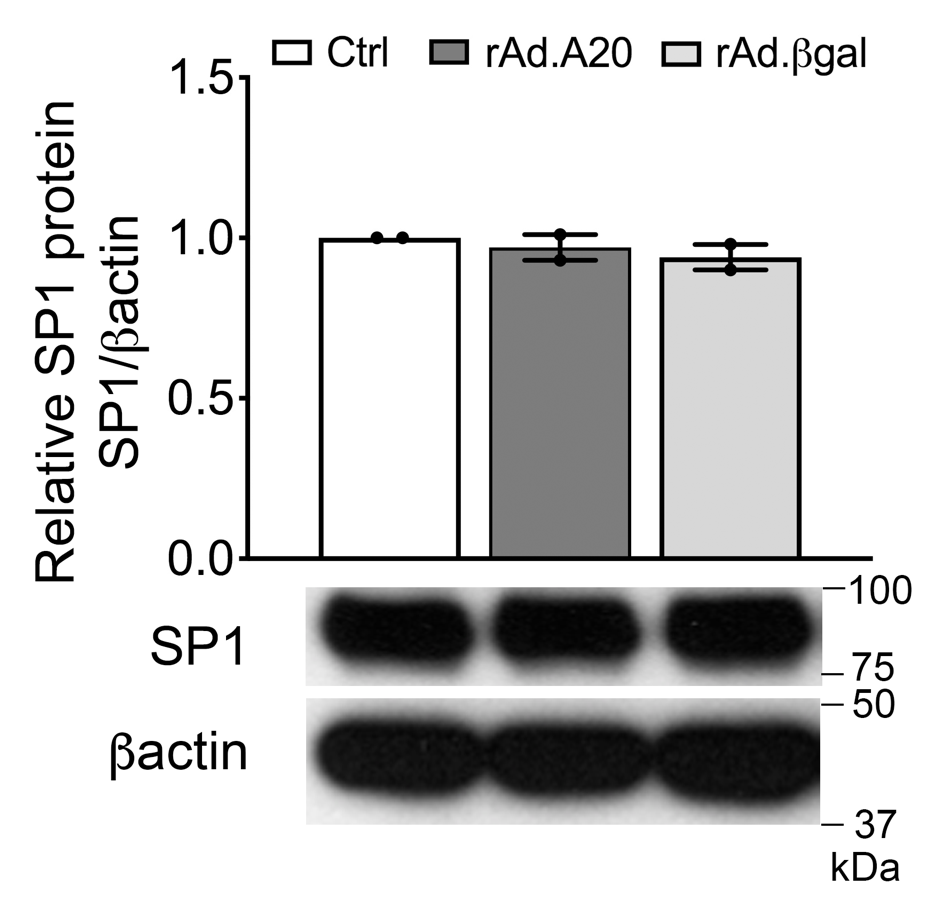


**Supplementary Figure 2**. **Overexpression of A20 in HCAEC does not affect protein levels of the transcription factor SP1.** Representative western blot analysis of SP1 in non-transduced HCAEC (Ctrl) and HCAEC transduced with rAd.A20 or control rAd.βgal at 100 MOI for 48 h. βactin was used to correct for loading and allow semi-quantitative evaluation of the data. Densitometry results are presented as fold change of Ctrl. Data are presented as mean ± SEM of 2 independent experiments.


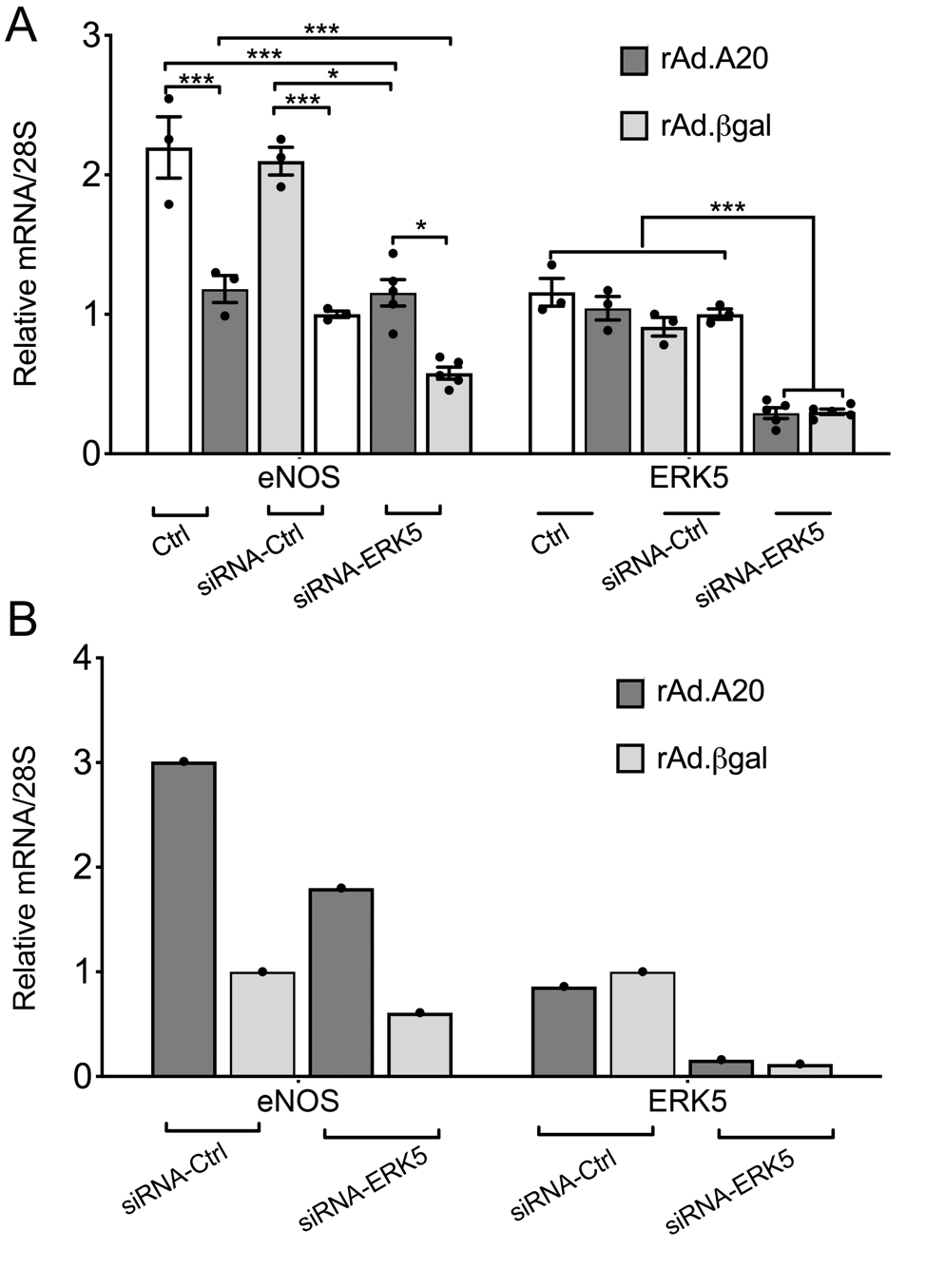


**Supplementary Figure 3. ERK5 is required for A20-mediated upregulation of eNOS transcription in HUVEC and HCAEC**. **A.** HUVEC were transfected with ERK5 silencing RNA (siRNA-ERK5) or control scrambled AllStars silencing RNA (siRNA-Ctrl) for 4 h, then transduced with rAd.A20 or control rAd.βgal at 100 MOI for an additional 24 h prior to mRNA extraction. eNOS and ERK5 mRNA levels were determined by qPCR. Graphs shown depict relative eNOS and ERK5 mRNA levels normalized by the 28S HKG. Data are presented as mean ± SEM fold change of siRNA-Ctrl and rAd.βgal-transduced cells (n= 3-5). **B.** Results were validated in HCAEC that underwent a similar transfection/transduction protocol. **p*<0.05, ****p*<0.001 as determined by two-way ANOVA followed by Bonferroni post-hoc test.

**
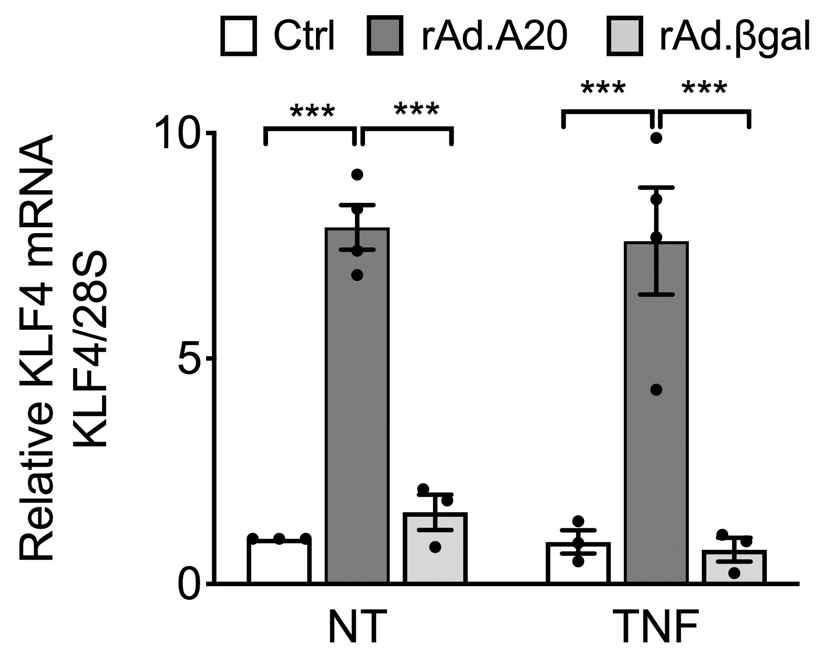
**

**Supplementary Figure 4. TNF does not affect A20-induced upregulation of KLF4 in HCAEC.** KLF4 mRNA levels were measured by qPCR in non-transduced HCAEC (Ctrl) and HCAEC transduced with rAd.A20 or control rAd.βgal at 100 MOI for 48 h prior to 24 h treatment with 200 U/mL of TNF. Graphs depict relative KLF4 mRNA levels normalized by the 28S, and expressed as mean ± SEM fold change of non-treated (NT) Ctrl cells (n=3-4). *** *p*<0.001, as determined by two-way ANOVA followed by Bonferroni pots-hoc test.


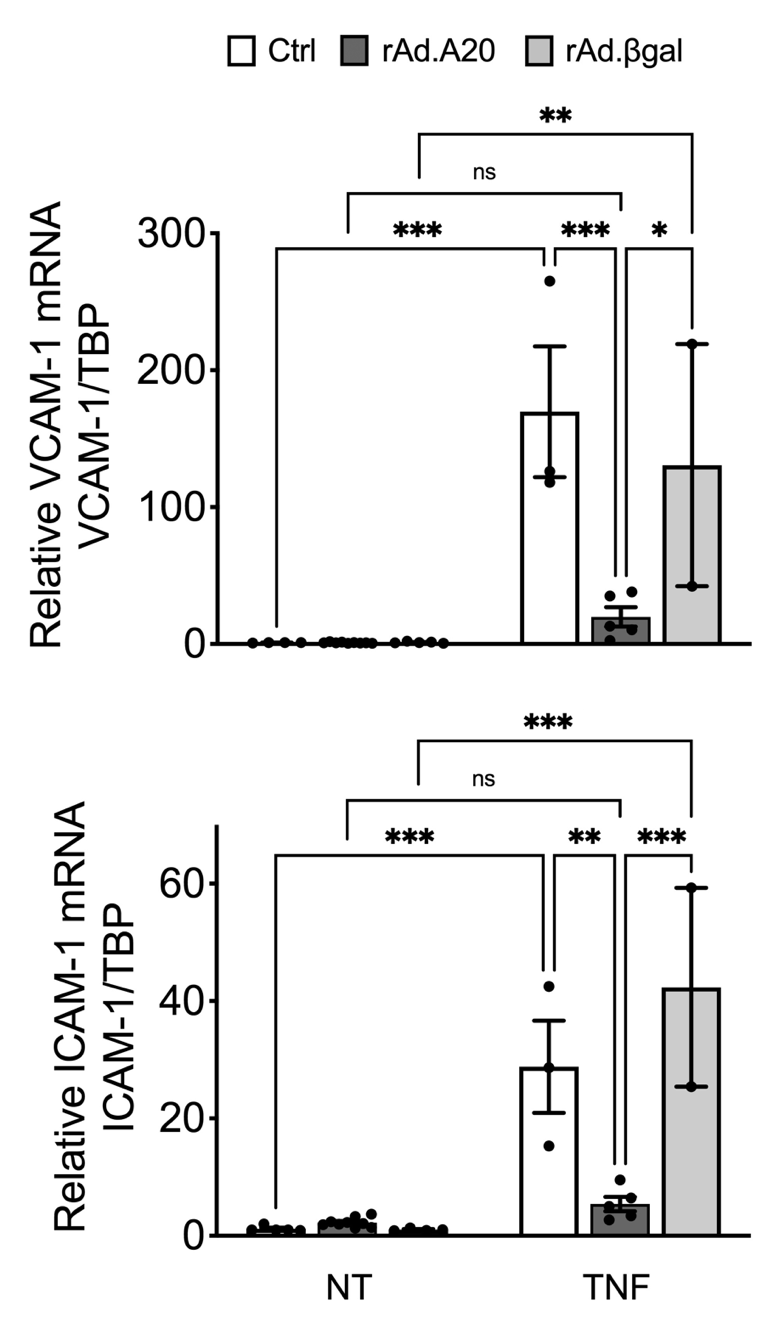


**Supplementary Figure 5. Overexpression of A20-induced in HCAEC inhibits TNF-mediated upregulation of VCAM-1 and ICAM-1.** VCAM-1 (A) and ICAM-1 (B) mRNA levels were measured by qPCR in non-transduced HCAEC (Ctrl) and HCAEC transduced with rAd.A20 or control rAd.βgal at 100 MOI for 48 h prior to 24 h treatment with 200 U/mL of TNF. Graphs depict relative VCAM-1 and ICAM-1 mRNA levels normalized by the TATA Box binding protein (TBP) HKG, and expressed as mean ± SEM fold change of non-treated (NT) Ctrl cells (n=2-6). *** *p*<0.001, ** *p*<0.01, * *p*<0.05, as determined by two-way ANOVA followed by Bonferroni pots-hoc test.


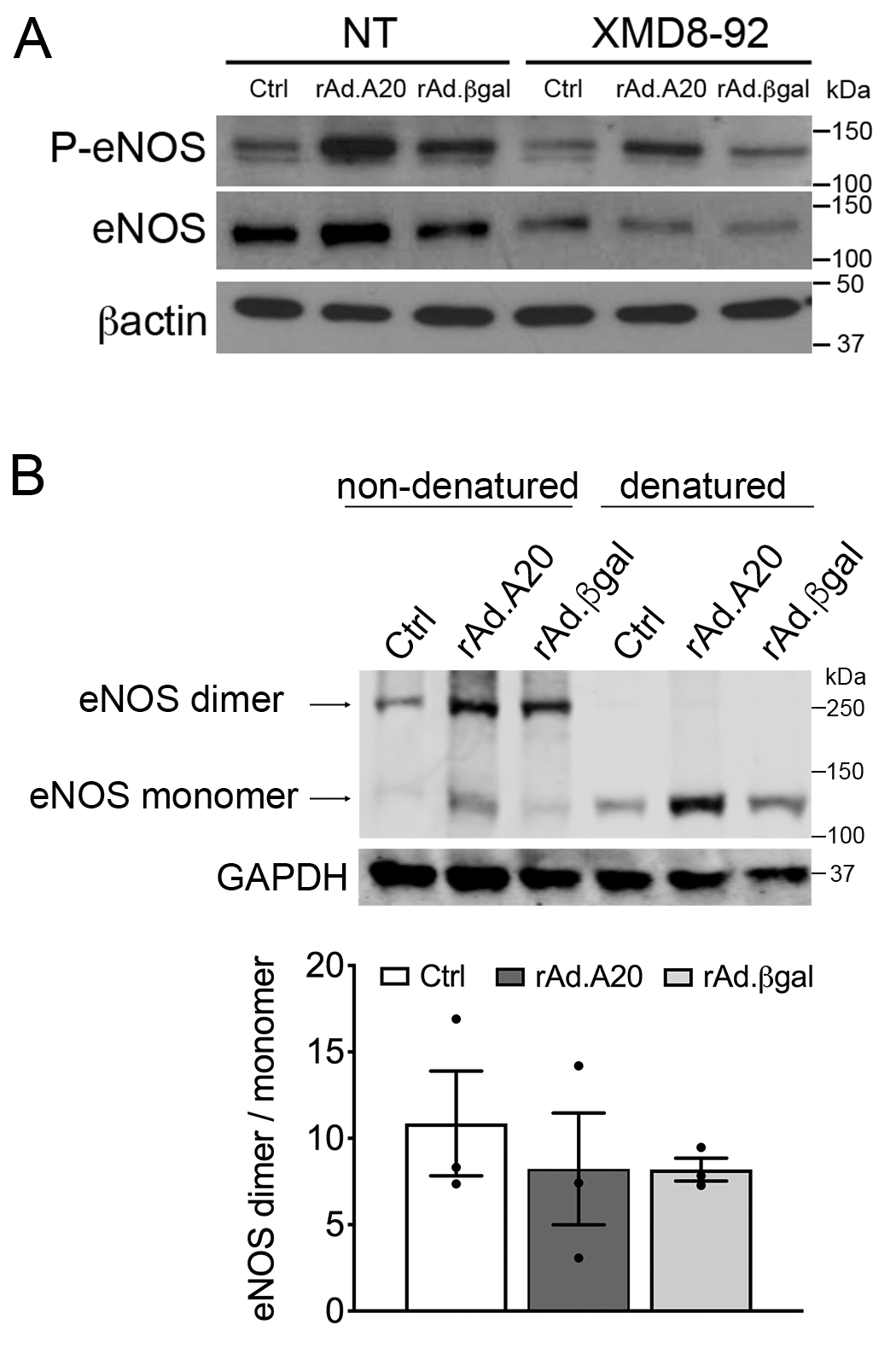


**Supplementary Figure 6.** **A20-induced eNOS phosphorylation does not depend on either ERK5 activation or changes in eNOS dimerization.** **A.** Representative western blot analysis of P-eNOS (Ser-1177) and total eNOS in non-transduced HCAEC (Ctrl) and HCAEC transduced with rAd.A20 or control rAd.βgal at 100 MOI, treated or not (NT) with the ERK5 inhibitor, XMD8-92 (10 μM). βactin was used to correct for loading. **B.** Representative western blot analysis of total eNOS in HCAEC non-transduced and transduced with rAd.A20 or control rAd.βgal at 100 MOI for 48h under non-reducing/non-denaturing conditions to preserve eNOS dimers. Samples processed under standard reducing and denaturing conditions were run in parallel for comparison. Probing for GAPDH was used to control for loading. Graph shows dimers over monomers ratio, as evaluated by densitometry (n=3).

**Supplementary Table 1: Primer sequences used for qPCR**

| **Gene name (ID)** | **Forward** | **Reverse** |
| --- | --- | --- |
| Homo sapiens Krüppel like factor 2 (KLF2) | CCACGATCCTCCTTGACGAG | CCGCAGACAGTACAAATTAAGGC |
| Homo sapiens Nitric oxide synthase 3 (NOS3) | GTTTGTCTGCGGCGATGTT | GCGTGAGCCCGAAAATGTC |
| Homo sapiens Peptidyl isomerase A (cyclophilin A) (PPIA) | AATGCTGGACCCAACAC | TCCACAATATTCATGCCTT |
| Homo sapiens 28S ribosomal RNA | CAGTTCTCTTGGGAATCCAG | TTCAGCAAAGGAGTCAATCCAC |
| Homo sapiens TNF alpha-induced protein 3 (TNFAIP3/A20) | CCCCATTGTTCTCGGCTAT | TCAAATCTTCCCCGGTCTC |
| Homo sapiens Extracellular signal-regulated kinase 5 (ERK5) | GCTGGCGCTCCTGGGCTGTCACC | GAGCCTGCCCCACCAAAGAAAGAT |
| Homo sapiens Krüppel like factor 4 (KLF4) | CAGCTTCACCTATCCGATCCG | GACTCCCTGCCATAGAGGAGG |
| Homo sapiens Endothelin 1 (END1) | AGAGTGTGTCTACTTCTGCCA | CTTCCAAGTCCATACGGAACAA |
| Homo sapiens Thrombomodulin (THDB) | ACCTTCCTCAATGCCAGTCAG | CGTCGCCGTTCAGTAGCAA |
| Homo sapiens Chemokine C-C motif ligand 2 (CCL2) | CTCATAGCAGCCACCTTCATT | CACAGCTCCCTTGGCCACAAT |
| Homo sapiens Intercellular Adhesion Molecule 1 (ICAM-1) | TGCCACCAATATGGGAAGGC | CCGAGCTCAAGTGTCTAAAG |
| Homo sapiens Vascular Cell Adhesion Molecule 1 (VCAM-1) | GGTGCTGCAAGTCAATGAGA | AAGATGGTCGTGATCCTTGG |
| Homo sapiens TATA-box binding protein (TBP) | CGTAATGGCTCTCATGTACCCT | ACCCTGCAACTCAACATCCATC |
